# Modulation of the immunity and inflammation by autophagy

**DOI:** 10.1002/mco2.311

**Published:** 2023-07-02

**Authors:** Ting Gan, Shu Qu, Hong Zhang, Xu‐jie Zhou

**Affiliations:** ^1^ Renal Division Peking University First Hospital Beijing China; ^2^ Peking University Institute of Nephrology Beijing China; ^3^ Key Laboratory of Renal Disease Ministry of Health of China Beijing China; ^4^ Key Laboratory of Chronic Kidney Disease Prevention and Treatment (Peking University) Ministry of Education Beijing China

**Keywords:** autophagy, immunity, inflammation, autoimmune diseases, pathogenesis

## Abstract

Autophagy, a highly conserved cellular self‐degradation pathway, has emerged with novel roles in the realms of immunity and inflammation. Genome‐wide association studies have unveiled a correlation between genetic variations in autophagy‐related genes and heightened susceptibility to autoimmune and inflammatory diseases. Subsequently, substantial progress has been made in unraveling the intricate involvement of autophagy in immunity and inflammation through functional studies. The autophagy pathway plays a crucial role in both innate and adaptive immunity, encompassing various key functions such as pathogen clearance, antigen processing and presentation, cytokine production, and lymphocyte differentiation and survival. Recent research has identified novel approaches in which the autophagy pathway and its associated proteins modulate the immune response, including noncanonical autophagy. This review provides an overview of the latest advancements in understanding the regulation of immunity and inflammation through autophagy. It summarizes the genetic associations between variants in autophagy‐related genes and a range of autoimmune and inflammatory diseases, while also examining studies utilizing transgenic animal models to uncover the in vivo functions of autophagy. Furthermore, the review delves into the mechanisms by which autophagy dysregulation contributes to the development of three common autoimmune and inflammatory diseases and highlights the potential for autophagy‐targeted therapies.

## Graphical abstract

This review presents an overview of the autophagy machinery and summarizes the roles of autophagy and autophagy proteins in regulating immunity and inflammation. The authors discuss how dysregulation of autophagy contributes to the pathogenesis of three common autoimmune and inflammatory diseases and emphasize autophagy as a promising therapeutic target.

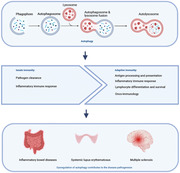

## 1 INTRODUCTION

Autophagy is a conserved process that facilitates the degradation and recycling of various cellular components by delivering them to lysosomes.^1^ Originally known as a mechanism for ensuring the quality of organelles and proteins, autophagy has also gained recognition as a means of survival under circumstances of limited energy or nutrient availability. Recent studies have expanded our knowledge regarding autophagy and revealed its role in immunity and inflammation. The emergence of this fresh outlook has been partly propelled by the linkages established between genetic variations in genes associated with autophagy and heightened vulnerability to autoimmune and inflammatory disorders. Following the discovery of the Thr300Ala variant in *ATG16L1* as a genetic predisposition for Crohn's disease (CD) via genome‐wide association studies (GWAS),^2^, ^3^ several research teams generated transgenic animal models to illustrate the crucial involvement of autophagy in maintaining intestinal epithelial homeostasis and defending against infections.^4^, ^5^, ^6^, ^7^, ^8^, ^9^, ^10^ Moreover, associations have been established between mutations in different genes related to autophagy and diverse autoimmune and inflammatory conditions,^11^, ^12^, ^13^, ^14^, ^15^ fueling significant enthusiasm in understanding the contribution of autophagy to the pathogenesis of these diseases. Considerable progress has been achieved in unraveling the functional aspects of autophagy in immunity and inflammation through the utilization of both in vitro and in vivo models.^16^, ^17^ By executing diverse functions such as pathogen clearance, antigen processing and presentation, cytokine production, as well as lymphocyte differentiation and survival, autophagy serves as a crucial bridge between the innate and adaptive immune systems. However, our comprehension of how autophagy influences immunity and inflammation is far from complete. Emerging studies have uncovered novel methods by which the autophagy pathway and autophagy proteins regulate immune responses, such as noncanonical autophagy.^18^, ^19^

This review highlights recent advancements in understanding the impact of autophagy on the regulation of immune responses and inflammatory processes. It presents the genetic links between variants in autophagy‐related genes (ATGs) and a range of autoimmune and inflammatory diseases. Additionally, it explores the in vivo function of autophagy through the utilization of transgenic animal models. Furthermore, this review delves into the underlying mechanisms by which autophagy dysregulation contributes to the pathogenesis of three prevalent autoimmune and inflammatory disorders: inflammatory bowel diseases (IBD), systemic lupus erythematosus (SLE), and multiple sclerosis (MS). Finally, it summarizes the findings of clinical trials using autophagy‐targeted drugs and biological agents and explores the prospects of therapies aimed at modulating autophagy.

## 2 AUTOPHAGY AND RELATED MOLECULAR PATHWAYS

**FIGURE 1:**
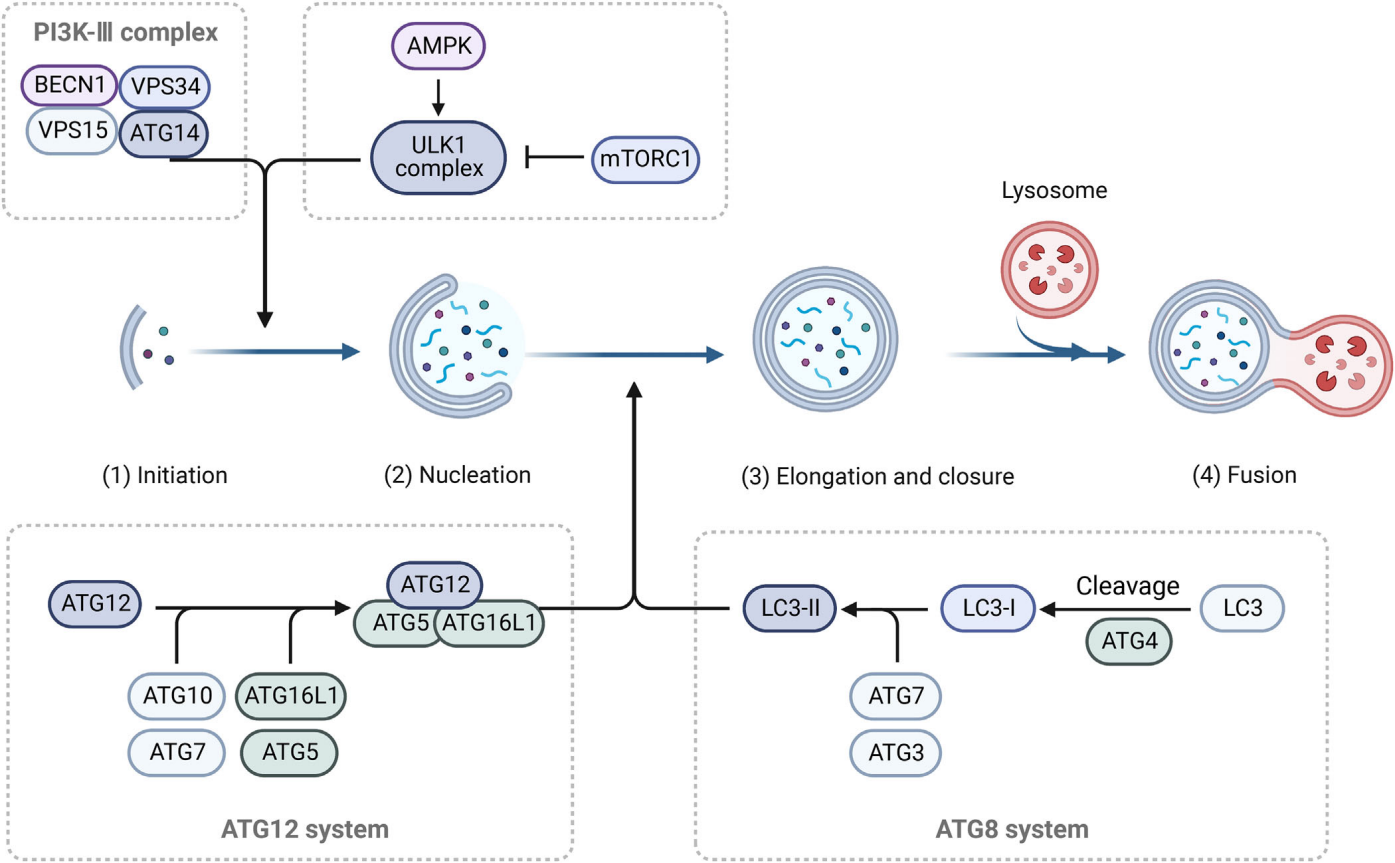
The process and molecular mechanism of autophagy. †Autophagy proceeds through a series of sequential phases, including autophagy initiation, nucleation of the autophagosome, elongation and closure of the autophagosome, and fusion of the autophagosome with lysosome. The process is mediated by a set of proteins encoded by autophagy‐related (ATG) genes.

Autophagy is a conservative mechanism of self‐degradation, which transports cellular materials to lysosomes for decomposition. The process of autophagy encompasses three distinct mechanisms: macroautophagy, microautophagy, and chaperone‐mediated autophagy, each employing unique strategies for transporting cargo to the lysosome.^20^, ^21^ Unless otherwise specified in this review, the extensively investigated form of autophagy, macroautophagy, will be predominantly referred to as such. The regulation of autophagy involves two key kinases: adenosine monophosphate (AMP) ‐activated protein kinase (AMPK), which monitors energy levels, and mammalian target of rapamycin (mTOR), which detects nutrient availability.^22^ The activation of AMPK, which is essential for regulating cellular energy metabolism, is triggered by alterations in energy status, such as an elevation in the AMP/ATP ratio or reduced glucose levels.^23^ Conversely, mTOR serves as a prominent controller of cell growth and protein synthesis, and its activation is induced by abundant nutrient availability, normoxic conditions, and elevated levels of growth factors.^22^ AMPK enhances autophagy by promoting the phosphorylation of ULK1, while mTOR inhibits autophagy by preventing ULK1 activation.^24^ Autophagy progresses through distinct stages encompassing autophagy initiation, autophagosome nucleation, autophagosome elongation and closure, as well as autophagosome fusion with lysosomes.^21^ The progression of this process is facilitated by a group of proteins encoded by ATG, as depicted in Figure 1. The intricate molecular mechanisms underlying autophagy are extensively discussed in other expert reviews.^25^

Although macro‐, micro‐, and chaperone‐mediated autophagy are all considered to be genuine forms of autophagy, as they transport cytoplasmic materials to lysosomes,^26^ recent research has revealed noncanonical autophagy pathways. These pathways are called noncanonical autophagy because they use distinct molecular pathways to process and conjugate microtubule‐associated protein light chain 3 (LC3).^27^ Noncanonical autophagy processes may specifically contribute to pathogen removal, surface molecule internalization, secretion/exocytosis, and phagocytosis.^26^ An exemplary instance is LC3‐associated phagocytosis (LAP),^18^ where specific constituents of the canonical autophagy machinery are engaged to facilitate the binding of LC3 to phagosomes with a single membrane.^28^, ^29^ LAP can be triggered by various surface receptors such as pattern recognition receptors (PRRs), immunoglobulin (Ig) receptors, and phosphatidylserine (PtdSer) receptors, which will be further discussed below.

## 3 AUTOPHAGY IN IMMUNITY AND INFLAMMATION

Although initially discovered for its role in degrading unwanted or damaged cytoplasmic material to maintain cellular homeostasis, recent studies also suggest that autophagy has broader functions in immunity and inflammation.^30^

Autophagy contributes to innate immunity by assisting in pathogen clearance and inflammation regulation. Two autophagy‐associated pathways, xenophagy and LAP, are utilized by innate immune phagocytes to promote degradation of microbes within lysosomes.^31^ Xenophagy, a typical type of autophagy, selectively captures intracellular pathogens and those in damaged intracellular vesicles into double‐membrane autophagosomes, which then undergoes degradation via the lysosomal pathway through autophagy.^32^ LAP, on the other hand, collaborates with canonical autophagy in cellular defense. Pathogenic microorganisms have been observed within LAP‐targeted, single‐membrane phagosomes, promoting phago‐lysosomal fusion and leading to enhanced microbial killing. Studies have revealed that the absence of ATGs, such as *Atg5*, renders mice more susceptible to infections and disrupts normal inflammatory responses.^33^ Autophagy is therefore critical to balance inflammatory response by timely removing exogenous and endogenous sources of inflammatory signals, such as microbes, damaged organelles, and aggregates. One of the major functions of the autophagy mechanism in regulating inflammation is to suppress inflammasome activation. The inflammasome functions as a receptor and sensor within the innate immune system, governing the activation of caspase‐1 and triggering the proteolytic cleavage of pro‐IL‐1β and pro‐IL‐18. This activation occurs upon the recognition of pathogen‐associated molecular patterns (PAMPs) or damage‐associated molecular patterns (DAMPs).^34^, ^35^ By eliminating endogenous inflammasome activators, such as damaged mitochondrial‐derived DAMPs, autophagy demonstrates its ability to dampen inflammasome activation.^36^ Additionally, autophagy can directly target inflammasome components and eliminate them by autophagic degradation. Autophagy plays an additional pivotal role in regulating inflammation by inhibiting the generation of type I interferon (IFN), which is mediated by cyclic guanosine monophosphate (GMP) –AMP synthase (cGAS) and stimulator of interferon genes (STING). The cGAS–STING pathway consists of the dsDNA/DNA hydrolysate sensor cGAS and the STING.^37^ Upon detection of dsDNA/dsRNA from exogenous and endogenous sources, cGAS catalyzes the production of cyclic GMP–AMP (cGAMP) using ATP and GTP. Subsequently, cGAMP binds to the active site of STING, initiating downstream signaling pathways and promoting the synthesis of type I IFN. Emerging evidence indicates that autophagy plays a role in mitigating excessive inflammation through downregulating the activation of the cGAS–STING pathway. The interaction between the autophagy‐related protein BECN1 and the cGAS DNA sensor has been identified as a key mechanism. This interaction promotes the degradation of cytoplasmic pathogen DNA through autophagy, effectively limiting the excessive production of inflammatory cytokines.^38^, ^39^ The deficiency of the ATG Atg9a has been shown to markedly increase the assembly of STING, resulting in aberrant activation of the innate immune response.^40^ Autophagic degradation of STING can attenuate STING‐directed type I IFN signaling activity. For instance, a newly identified autophagy receptor CCDC50 delivers K63‐polyubiquitinated STING to the autolysosome for degradation.^41^ Furthermore, it has been observed that the small‐molecule chaperone UXT facilitates the autophagic degradation of STING1 by promoting its interaction with SQSTM1, a process that contributes to the regulation of STING1 levels.^19^, ^42^ These findings emphasize the essential role of autophagy in modulating the activation of STING to maintain immune homeostasis.

Autophagy plays a pivotal role in bridging innate and adaptive immunity by facilitating antigen presentation. Recent studies have provided emerging evidence that autophagy plays a facilitating role in the presentation of antigens on major histocompatibility complex (MHC) class II molecules.^43^ Two autophagy‐related mechanisms exist in antigen‐presenting cells (APCs) for the presentation of antigens on MHC class II molecules. The first mechanism involves the capture and delivery of extracellular antigens to the autophagosome, where MHC class II molecules are subsequently recruited to the phagolysosome. This process generates immunogenic peptides that engage with CD4+ T cells. The second mechanism entails the utilization of noncanonical autophagy LAP, wherein specific receptors like Toll‐like receptors (TLRs) recognize the cargo, resulting in the conjugation of LC3 to single‐membrane phagosomes and the subsequent formation of LAPosomes.^19^ The fusion between LAPosomes and lysosomes facilitates the efficient degradation of cargo and the generation of fragments that can be loaded onto MHC class II molecules, thereby triggering the activation of CD4+ T cells. Furthermore, autophagy plays a role in directing cytosolic proteins for degradation in lysosomes, thereby enhancing the presentation of intracellular antigens on MHC class II molecules and facilitating robust CD4+ T cell responses. It is also crucial to consider the role of autophagy in the context of endogenous MHC class II loading during thymic selection, as it plays a vital role in shaping the T cell repertoire.^44^ Considerable autophagic activity is observed in thymic epithelial cells (TECs), which plays a crucial role in the establishment of self‐tolerance in CD4+ T cells by presenting self‐antigens on MHC class II molecules during their development.^16^ Altered selection of specific MHC‐II restricted T cells and the development of multi‐organ inflammation are observed in TECs lacking *Atg5*.^45^, ^46^ These findings highlight the crucial involvement of autophagy in inducing tolerance during the development of the T cell repertoire.^47^ In addition to its role in antigen presentation, autophagy also plays a crucial role in modulating the development and maintenance of immune cells, thus contributing to adaptive immunity. For instance, targeted deletion of *Atg5* in distinct subsets of immune cells in murine models has been shown to result in notable impairments in various aspects of immune function, such as compromised B lymphocyte maturation, impaired differentiation of plasma cells, diminished T cell survival, and reduced T cell proliferation.^48^, ^49^, ^50^

In addition to its aforementioned immunomodulatory functions, autophagy's role in onco‐immunology is also increasingly recognized. The involvement of autophagy in tumor development is influenced by multiple variables, such as the specific tumor subtype, stage of progression, and the characteristics of the tumor microenvironment. Autophagy plays a critical role in maintaining homeostasis, activating, differentiating, and promoting the survival of immune cells, which in turn can have both promotive and inhibitory effects on tumor development. Thus, autophagy functions as a double‐edged sword in the context of tumorigenesis. Since the topic of autophagy and tumor immunity has been covered comprehensively in recent reviews,^51^, ^52^, ^53^, ^54^ we will focus on a few studies of notable significance to discuss the impact of autophagy on tumor immunity. To begin, autophagy plays a role in regulating antigen processing and presentation within tumor cells. MHC class I (MHC‐I) molecules are crucial for antitumor adaptive immunity as they facilitate the presentation of endogenous antigens to CD8+ T cells.^55^ Impaired MHC‐I expression in tumor cells can hinder antigen presentation and enable immune evasion. Recent investigations have suggested that autophagy may contribute to immune evasion in pancreatic cancer by participating in the degradation of MHC‐I molecules.^56^ In pancreatic ductal adenocarcinoma (PDAC) cells, lysosomes were found to be enriched with MHC‐I, while its expression on the cell surface was reduced. Researchers discovered that the autophagy cargo receptor NBR1 facilitates the transport of MHC‐I molecules to lysosomes for degradation via an autophagy‐dependent pathway.^56^ Inhibition of autophagy restores surface MHC‐I levels, leading to increased antigen presentation, enhanced proliferation and activation of CD8+ T cells, and augmented tumor cell elimination. Additionally, autophagy inhibition through ULK1 targeting has been demonstrated to overcome impaired antigen presentation and restore antitumor immunity in lung cancer with LKB1 mutation.^57^ Therefore, interfering with autophagy in tumor cells may provide an effective way to promote an antitumor T‐cell response. Furthermore, recent discoveries have highlighted the involvement of LAP in the engulfment of apoptotic tumor cells. During tumor progression, the heightened rate of cell proliferation often coincides with an elevated incidence of cell death. Moreover, anticancer treatments can induce cell death in tumors. Therefore, the presence of numerous dying or dead cells in the tumor microenvironment provides an abundant supply of pre‐existing substrates for professional phagocytic cells.^58^ LAP utilizes autophagy machinery components to facilitate optimal maturation of phagocytic cells and degradation of ingested cargo. LAP can promote clearance of dying or dead cells by interacting with the surface receptor T‐cell Ig mucin protein 4 (TIM‐4) on macrophages.^59^ New research findings suggest that LAP plays a significant role in the engulfment of dying and deceased cells by bone marrow macrophages within the microenvironment of acute myeloid leukemia (AML).^60^ Researchers found that apoptotic bodies from AML cells were taken up by bone marrow macrophages via LAP, followed by mtDNA stimulation of STING to enhance phagocytosis and inhibit AML cell growth.^59^ Therefore, AML disease progression is expedited in mice with deficiencies in LAP. However, this macrophage function associated with AML contrasts with the immunosuppressive role of LAP in solid tumors. Researchers have noted that in mice with solid tumors like melanoma and lung cancer, LAP present in myeloid cells facilitated the growth of the tumors.^61^ At the mechanistic level, LAP serves as a regulatory mechanism in steering the polarization of tumor‐associated macrophages (TAMs) toward an immunosuppressive state, as demonstrated by recent studies.^61^ Additionally, after engulfing apoptotic tumor cells, LAP inhibits the production of type I IFN in TAMs. Therefore, it suppresses T cell function and enhances tumor tolerance. These findings emphasize that the effect of LAP is contingent on various factors, including cancer type and stage. Further study is warranted to shed light on the underlying reasons for these discrepancies.

## 4 AUTOPHAGY IN AUTOIMMUNE AND INFLAMMATORY DISEASES

The association between mutations in ATGs and heightened susceptibility to autoimmune and inflammatory diseases has piqued interest in the involvement of autophagy in disease mechanisms. Nevertheless, as indicated in Table 1, the majority of genetic variations are found within noncoding regions, with only a limited portion of the predicted heritability attributable to functional variations. As a result, it is challenging to translate genetic variation information into mechanistic insights. Nonetheless, transgenic animals are useful tools for improving our understanding of disease etiology. In particular, when animal models with functional variations are shown to alter gene functions, it can provide strong evidence for the gene's contribution to disease pathogenesis. The subsequent sections will focus on three common autoimmune and inflammatory diseases and examine how autophagy dysregulation contributes to disease development, primarily drawing from evidence obtained through transgenic animal models.

**TABLE 1:** The list of variants in autophagy‐related genes associated with the susceptibility to several autoimmune and inflammatory diseases.

Disease	Gene	Chromosome	SNP	Reference	Alternate	Functional annotation	OR	*p* Value	Study strategy	References
IBD	*ATG16L1*	2	rs2241880 (T300A)	A	G	missense_variant	1.32	3.00E−06	GWAS & fine‐mapping	^62^
							1.45	1.00E−13	GWAS & fine‐mapping	^63^
							1.32	1.00E−12	GWAS & fine‐mapping	^64^
			rs3828309	A	G	intron_variant	1.25	2.00E−32	GWAS meta‐analysis	^65^
			rs12994997	G	A	intron_variant	1.23	4.00E−70	GWAS meta‐analysis	^66^
			rs10210302	C	T	intron_variant	1.19	5.00E−14	GWAS & fine‐mapping	^67^
	*IRGM*	5	rs11747270	A	G	intron_variant	1.33	3.00E−16	GWAS meta‐analysis	^65^
			rs1000113	C	T	intron_variant	1.54	3.00E−07	GWAS & fine‐mapping	^67^
			rs11741861	A	G	intron_variant	1.33	6.00E−44	GWAS meta‐analysis	^68^
			rs13361189	T	C	intergenic_variant	1.38	2.00E−10	Candidate gene	^69^
			rs7714584	A	G	intron_variant	1.37	8.00E−19	GWAS meta‐analysis	^70^
	*ATG16L2*	11	rs11235604	C	T	missense_variant	1.61	2.44E−12	GWAS & fine‐mapping	^71^
	*LRRK2*	12	rs11175593	C	T	non_coding_transcript_exon_variant	1.54	3.00E−10	GWAS meta‐analysis	^65^
			rs4768236	C	A	intron_variant	1.12	4.00E−21	GWAS meta‐analysis	^68^
	*ULK1*	12	rs12303764	T	G	intron_variant	1.32	2.00E−04	Candidate gene	^72^
SLE	*ATG7*	3	rs11706903	C	A	intron_variant	1.26	1.12E−04	GWAS pathway analysis	^73^
	*IRGM*	5	rs10065172	C	T	synonymous_variant	1.15	1.50E−02	GWAS pathway analysis	^73^
			rs13361189	T	C	5' of *IRGM*	1.13	3.00E−02	GWAS pathway analysis	^73^
	*ATG5*	6	rs548234	C	T	3' of *ATG5*	1.25	5.00E−12	GWAS & fine‐mapping	^11^
			rs6937876	G	A	3' of *ATG5*	1.70	1.40E−02	Candidate gene	^73^
			rs2245214	C	G	intron_variant	1.15	1.20E−05	Candidate gene	^74^
	*ATG16L2*	11	rs11235604	C	T	missense_variant	0.76	1.90E−09	GWAS & fine‐mapping	^12^
	*DRAM1*	12	rs4622329	G	A	intron_variant	1.12	4.00E−15	GWAS meta‐analysis	^13^
							1.19	9.00E−12	GWAS meta‐analysis	^75^
	*CLEC16A*	16	rs34361002	T	TAA	intron_variant	1.14	1.00E−17	GWAS meta‐analysis	^13^
			rs12599402	T	C	intron_variant	1.28	5.00E−07	GWAS meta‐analysis	^75^
			rs7200786	A	G	intron_variant	1.15	2.00E−08	GWAS meta‐analysis	^76^
			rs9652601	G	A	intron_variant	1.21	7.00E−17	GWAS meta‐analysis	^76^
							1.17	4.00E−07	GWAS meta‐analysis	^77^
							1.19	6.00E−13	GWAS meta‐analysis	^78^
			rs2041670	G	A	intron_variant	1.18	2.00E−16	GWAS meta‐analysis	^78^
			rs8054198	C	T	TF_binding_site_variant	2.78	2.00E−08	GWAS meta‐analysis	^78^
	*MAP1LC3B*	16	rs933717	T	C	intron_variant	0.13	2.36E−10	GWAS pathway analysis	^79^
	*MTMR3*	22	rs9983	G	A	3_prime_UTR_variant	1.61	2.07E−03	GWAS shared genetics	^80^
MS	*CALCOCO2*	17	rs550510	G	A	missense_variant	0.65	7.00E−03	Candidate gene	^81^
RA	*ATG16L1*	2	rs2241880	A	G	missense_variant	1.32	2.00E−02	Candidate gene	^82^
	*ATG5*	6	rs62422878	C	T	intron_variant	1.11	4.00E−09	GWAS meta‐analysis	^14^
			rs9372120	T	G	intron_variant	0.11	1.00E−07	GWAS meta‐analysis	^15^
							1.11	8.00E−10	GWAS meta‐analysis	^83^
	*UVRAG*	11	rs7111334	C	T	intron_variant	0.25	1.50E−02	Candidate gene	^84^
	*GABARAPL3*	15	rs6496667	C	A	5' of *GABARAPL3*	1.09	1.00E−06	GWAS meta‐analysis	^85^
Psoriasis	*ATG16L1*	2	rs13005285	T	G	intron_variant	1.32	2.40E−02	Candidate gene	^86^

Data were collected from GWAS catalog database (https://www.ebi.ac.uk/gwas/); IBD, inflammatory bowel diseases; SLE, systemic lupus erythematosus; MS, multiple sclerosis; RA, rheumatoid arthritis; GWAS, genome‐wide association study; SNP, single‐nucleotide polymorphism; OR, odds ratio.

### 4.1 Inflammatory bowel diseases

IBD, encompassing CD and ulcerative colitis, manifests as a chronic gastrointestinal disorder marked by severe inflammation and mucosal damage.^87^ The exact mechanism of IBD remains unclear, but potential causes such as environmental factors, infectious agents, and genetic susceptibility have been proposed.^88^ Many genetic variants in ATG genes have been identified as influential factors in the onset of IBD and one of these variants is a missense mutation (rs2241880, Thr300Ala) located in the autophagy gene *ATG16L1*. This discovery has generated significant interest in unraveling the role of autophagy in the context of IBD. Moreover, GWAS have revealed additional single‐nucleotide polymorphisms (SNPs) in ATG genes that exhibit a strong association with IBD, as shown in Table 1.

**FIGURE 2:**
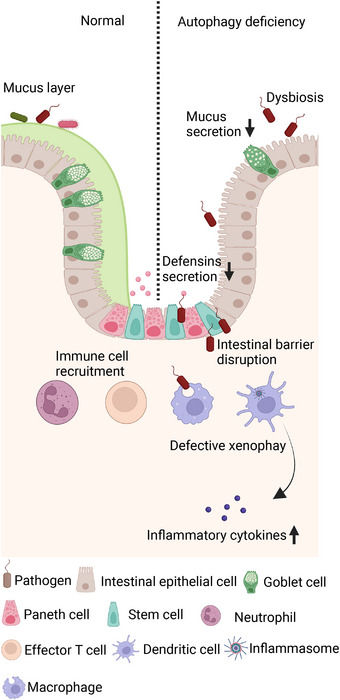
The roles of autophagy deficiency in pathogenesis of inflammatory bowel diseases (IBD). †Defective autophagy can affect gut microbiota composition, disrupt intestinal epithelial homeostasis, and amplify intestinal inflammation in IBD.

Considering that most of the evidence linking autophagy‐related genetic variants to IBD is derived from functional studies utilizing the *ATG16L1* T300A variant, our discussion will focus primarily on *ATG16L1*, supplemented by other ATGs. The T300A variant (Thr300Ala) causes the substitution of the evolutionarily conserved polar threonine residue at position 300 of the WD repeat domain in ATG16L1 with a nonpolar alanine. During the process of autophagosome formation, the ATG16L1 protein engages in interactions with the ATG12–ATG5 conjugate, leading to the formation of a large molecular complex. This complex facilitates the lipidation of LC3/ATG8, thereby contributing to autophagosome development.^89^, ^90^ Mice with functional defects in *Atg16l1* display dysregulated autophagy and intestinal homeostasis, and the relevant mechanisms are summarized in Figure 2.

#### 4.1.1 Intestinal epithelial homeostasis

In a healthy state, the intestinal epithelial cells (IECs) establish a strong mucosal barrier capable of withstanding intestinal pathogens. Additionally, specialized epithelial cells, including Paneth and goblet cells, secrete defensins and mucus to limit bacterial adhesion and infiltration.^91^ However, patients with IBD often exhibit defects in maintaining mucosal barrier stability, which leads to dysregulated mucosal immune responses to gut microbiota. Xenophagy is essential for eliminating intracellular pathogens, and autophagy‐associated risk variants have been linked to defective autophagy and impaired intracellular bacterial clearance.^92^ Conway et al.^4^ generated mice lacking *Atg16l1* in epithelial cells and found that autophagy‐deficient mice had systemic translocation of bacteria and an exaggerated inflammatory response compared with their wild‐type counterparts. Under normal conditions, autophagosomes can engulf invaded *S Typhimurium* in IECs. However, mice with *Atg16l1* deletion in IECs showed cell‐specific disruptions of autophagy and failed to control the spread of intestinal pathogens. In addition, these mice displayed abnormal morphology of Paneth cells, specialized epithelial cells that play a crucial role in the secretion of antimicrobial peptides.^93^, ^94^ In fact, IECs deficient in other ATGs, such as *Irgm1* ^95^ and *Atg4b*,^96^ also showed marked Paneth cell alterations. Moreover, autophagy dysfunction in *Atg16l1* ^T300A/T300A^ mice caused a defect in mucin secretion in their goblet cells.^5^ Thus, impaired autophagy may compromise the release of antimicrobial peptides and mucin, leading to heightened vulnerability of the intestinal epithelial barrier to microbial infections. These observations underscore the crucial role of autophagy in preserving the equilibrium of the intestinal immune system through efficient elimination of harmful pathogens in the gut.

#### 4.1.2 Inflammatory immune response

IBD are chronic gastrointestinal inflammatory conditions, and autophagy has been underscored in recent research as a vital mechanism for regulating inflammatory responses.^6^, ^97^ Inflammasomes are typically activated upon stimulation by PAMPs or DAMPs to safeguard host cells against microbial infections. However, prolonged hyperactivation of inflammasomes can lead to excessive cytokine production and severe inflammatory diseases, including IBD. Therefore, precise control of inflammasome activation is indispensable in upholding intestinal equilibrium. Autophagy functions as an innate cellular defense mechanism by capturing the invaded pathogens, thereby preventing the recognition of PRRs and downstream inflammasome signaling. Autophagy defects have been reported to cause excessive inflammasome activation and abnormal inflammation. Saitoh et al.^7^ showed that *Atg16l1*‐deficient macrophages exhibited heightened production of the proinflammatory cytokines IL‐1β and IL‐18 upon stimulation with lipopolysaccharide, owing to the activation of caspase‐1.^98^ Additionally, mice harboring the *Atg16l1* T300A variant, introduced via knock‐in, exhibited defective antibacterial autophagy and an increased cytokine response.^8^, ^9^ This phenomenon arises due to the increased susceptibility of the variant Atg16L1 T300A protein to cleavage by caspase 3 and caspase 7, as compared with the wild‐type protein. Consequently, this cleavage event results in reduced levels of functional ATG16L1 and perturbed cytokine signaling pathways. On the other hand, elevated inflammatory cytokines would accelerate the apoptosis of autophagy‐deficient IECs, resulting in impaired barrier function and exacerbated pathology.^10^ Autophagy in the epithelium has been shown to reduce TNF‐induced apoptosis and limit intestinal inflammation. Therefore, functional autophagy is crucial for tightly regulating intestinal inflammation.

#### 4.1.3 Gut microbiota

The gut microbiota comprises trillions of bacteria inhabiting the mammalian intestinal lumen, and their interaction with the host is essential for maintaining physiological metabolism and intestinal immune equilibrium. The importance of autophagy in regulating the gut microbiota has been underscored by recent investigations. By eliminating intracellular pathogens, autophagy restricts the replication and dissemination of pathogenic microorganisms. In addition, autophagy helps regulate the gut microbiota composition by maintaining mucosal barrier stability. It is involved in secreting antimicrobial peptides and mucus into the intestinal lumen, which safeguards against pathogen invasion and infection. Dysfunctional autophagy has been associated with dysbiosis of the gut microbiota, which heightens the susceptibility to developing IBD.^99^ Specifically, mice with gut‐specific *Atg5* knockout display an altered gut microbiota composition and structure, characterized by an upsurge of proinflammatory bacteria and a decrease in alpha diversity.^100^, ^101^ Mice specifically lacking *Atg7* in colonic epithelial cells also exhibit comparable outcomes.^102^ Additionally, mice carrying the *Atg16l1* ^T300A/T300A^ mutation exhibit an altered gut microbiota composition, characterized by a notable increase in the prevalence of bacteria linked to IBD, such as *Ruminococcaceae*.^5^, ^103^ These discoveries emphasize the significance of autophagy in maintaining gut microflora homeostasis.

### 4.2 Systemic lupus erythematosus

**FIGURE 3:**
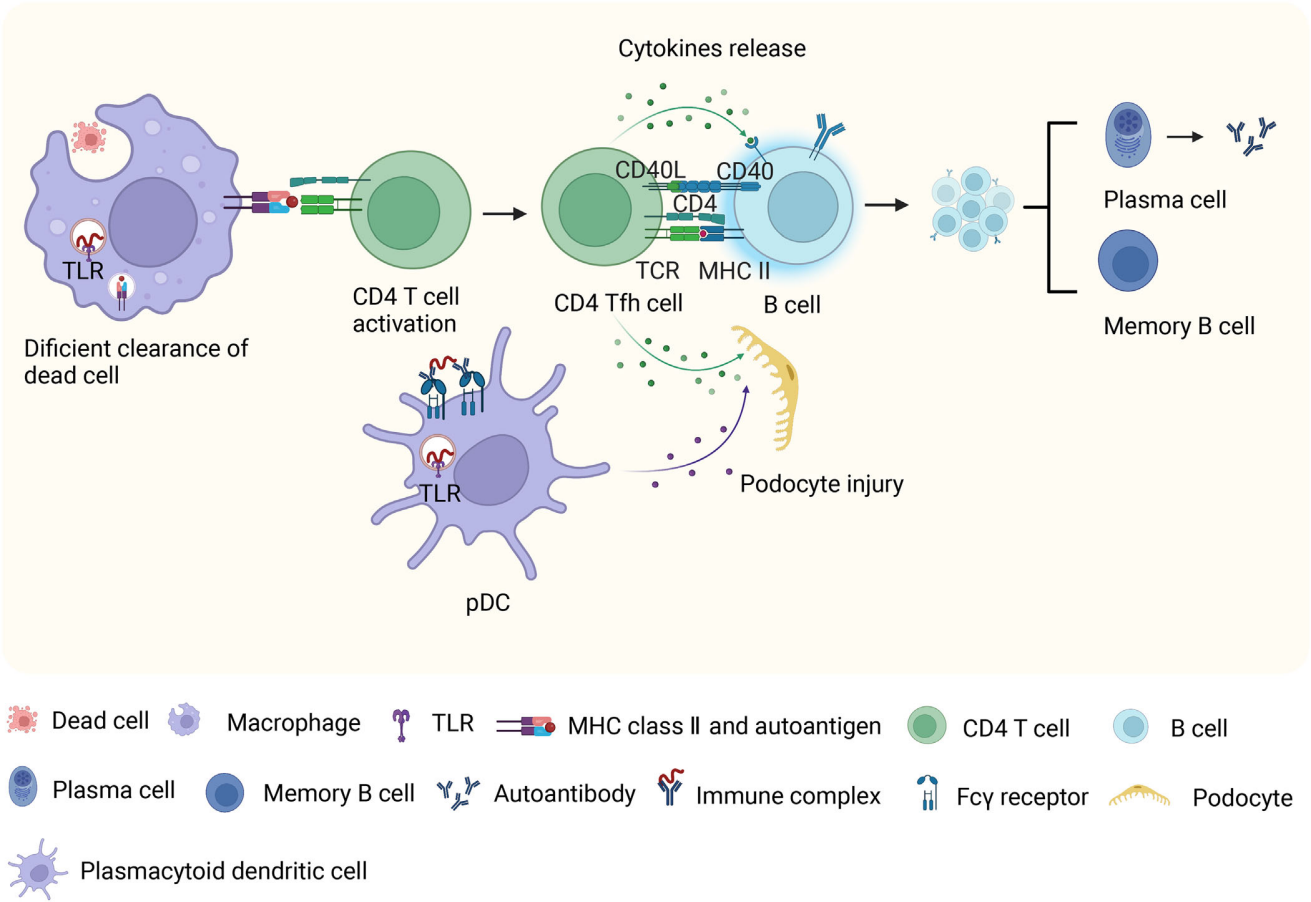
The roles of autophagy dysregulation in the pathogenesis of systemic lupus erythematosus (SLE)/lupus nephritis (LN). †A deficiency in autophagy or noncanonical autophagy can result in defective processing and degradation of dead cells, overproduction of inflammatory cytokines, and autoreactive B cell activation, leading to increased proliferation and differentiation of B cell. Plasmacytoid dendritic cells (pDCs) can trigger the production of type I interferon by internalizing immune complexes via noncanonical autophagy. In the context of LN, autophagy can protect podocytes from injury, such as pathogenic autoantibodies, and interferon‐α.

SLE is distinguished by an excessive immune response and the breakdown of immune tolerance toward self‐antigens.^104^ As shown in Table 1, the results of GWAS studies have yielded valuable insights into the link between genetic variations in ATGs and SLE susceptibility.^74^, ^75^, ^105^, ^106^ Further research involving cell biology and animal models has elucidated the functional mechanisms through which autophagy contributes to lupus pathogenesis. It is worth noting that LAP has also been shown to regulate the immune response in SLE (refer to Figure 3).

#### 4.2.1 The exposure of autoantigens

One of the key factors in the development of SLE is the imbalance between the accumulation of material from dead cells and the disposal of apoptotic debris, which contributes to increased autoantigen exposure. Under normal circumstances, professional phagocytes like macrophages clear apoptotic debris, which remains sequestered from the immune system. However, impaired clearance of dead cell debris can activate nucleic acid recognition receptors, triggering the production of proinflammatory cytokines. Recent research has emphasized the crucial function of LAP, a distinct form of noncanonical autophagy, in the efficient removal of dead cells.^107^, ^108^ LAP facilitates phagosome maturation (i.e., LAPosomes) and the digestion of extracellular cargo using only a subset of autophagy machinery components.^109^ Specifically, NADPH oxidase‐2 (NOX2) and Rubicon are indispensable components of LAP. Macrophages can trigger LAP and mediate subsequent efficient degradation of engulfed dead cells when the TIM4 receptor recognizes and engages with the “eat me” signal PtdSer expressed on dead cells. Uptake and degradation of dead cells are crucial for preventing an unwanted autoimmune response. In the absence of LAP, macrophages may have defects in the degradation of dead cells, resulting in an increase in the production of proinflammatory cytokines.^107^ Additionally, mice with deficiencies in LAP pathway components, such as *Cybb* and *Ncf1*, displayed deficient uptake and degradation of necrotic material, increased production of inflammatory mediators, or exacerbated lupus.^110^, ^111^

#### 4.2.2 The differentiation and survival of B cells

One prominent characteristic of SLE is the generation of autoantibodies by self‐reactive B cells.^112^ Extensive investigation underscores the importance of autophagy in the development of SLE by influencing B cell differentiation and the generation of autoantibodies.^113^, ^114^ In individuals with SLE, B cells exhibit elevated levels of autophagy compared with healthy individuals, and there exists a positive association between autophagosome density and disease activity as measured by the SLE Disease Activity Index (SLEDAI).^113^ Deleting the negative autophagy regulator *Rubcn* has been found to provide survival advantages for SLE‐prone mice, including B6.Sle1.Yaa and MRL.Fas^lpr^ mice, by reducing autoantibody production and protecting them from renal disease.^115^ *Rubcn* promotes autoimmunity in murine lupus by activating autoreactive B cells, promoting autoreactive germinal center reactions and plasmablast response. Autophagy also plays a critical role in the differentiation of plasma cells and the survival of memory B cells.^49^, ^116^, ^117^, ^118^ Deficiency in *Atg5* or *Atg7* impairs the terminal differentiation of B cells into plasma cells.^49^, ^113^ Moreover, autophagy is essential for maintaining the homeostasis of plasma cells, as these cells produce a large quantity of Igs, making them susceptible to severe endoplasmic reticulum stress, oxidative stress, and proteasome stress.^116^, ^119^ Plasma cells deficient in *Atg5* are more vulnerable to cell death because they lack the protective effects provided by autophagy.^116^, ^117^ Modulating autophagy in B cells represents a potential strategy for controlling the production of autoantibodies, as the maintenance of memory cells is vital for long‐term humoral autoimmunity, and autophagy has been identified as a crucial player in this process.^118^

#### 4.2.3 Inflammatory immune response

Exposure of the body to autoantigens containing nucleic acid can trigger an immune response, leading to the production of autoantibodies. The subsequent interaction between these autoantibodies and autoantigens can result in the formation of immune complexes, which possess the ability to initiate inflammation and cause tissue damage. Plasmacytoid dendritic cells (pDCs) take up immune complexes by binding to Fcγ receptors (FcγRs) and transport them to endosomal compartments where TLR7 and TLR9 are located.^120^ This process leads to the overproduction of type I IFNs by pDCs.^121^, ^122^ LAP assists in transporting DNA‐immune complexes to TLR9, initiating signaling from FcγRs leading to type I IFN production.^123^ Hayashi et al.^124^ found that the recruitment of the IKKα complex to LC3‐containing LAPosomes is necessary for TLR9 signaling and type I IFN production. Autophagy is essential for the development of TLR7‐mediated SLE.^125^ In a comparison of SLE development in *Tlr7.1* transgenic mice with and without B cell‐specific *Atg5* knockout, researchers observed that *Tlr7.1* transgenic *Atg5* knockout mice did not exhibit an IFN‐α signature in their serum.^125^ This finding suggests that autophagy participates in the delivery of RNA ligands to the endosomes, thereby mediating the activation of autoreactive B cells. Additionally, dendritic cell‐specific *Atg5* knockout mice also exhibited reduced IFNα and increased lifespan,^126^ indicating the functional roles of autophagy in inflammation.

#### 4.2.4 Response of kidney resident cells

Lupus nephritis (LN) is a serious complication and a primary contributor to mortality among SLE patients.^127^ While the exact function of autophagy in LN remains uncertain, its protective role in podocytes has been established.^22^ Autophagosomes in podocytes have been observed in both MRL^lpr/lpr^ mice suffering from nephritis and in renal biopsies from LN patients, signifying the activation and participation of autophagy in the development of LN.^128^ When podocytes are exposed to pathogenic autoantibodies and IFN‐α, activated autophagy is negatively associated with indicators of podocyte injury such as podocyte apoptosis, podocin derangement, impaired cell migration, and increased cell permeability. Conversely, inhibition of autophagy worsens podocyte damage. It can be inferred from this evidence that autophagy is a crucial mechanism for protecting podocytes from injury.^128^, ^129^ The significance of autophagy and inflammasome signaling interactions in the regulation of podocyte injury has also been emphasized by recent studies.^129^, ^130^, ^131^ Autophagy can capture and degrade inflammasomes via inflammasome ubiquitination, thereby controlling excessive inflammasome activation.^132^ The expression of NLR family pyrin domain‐containing 3 (NLRP3) is significantly increased in the podocytes of patients with LN, and this upregulation is linked to podocyte injury.^129^ In an intervention study, the augmentation of autophagy has been discovered to mitigate podocyte injury through the elimination of the NLRP3 inflammasome, whereas inhibition of autophagy can exacerbate podocyte injury. These results indicate the unique value of autophagy of the kidney resident cells in protecting against LN.

### 4.3 Multiple sclerosis

**FIGURE 4:**
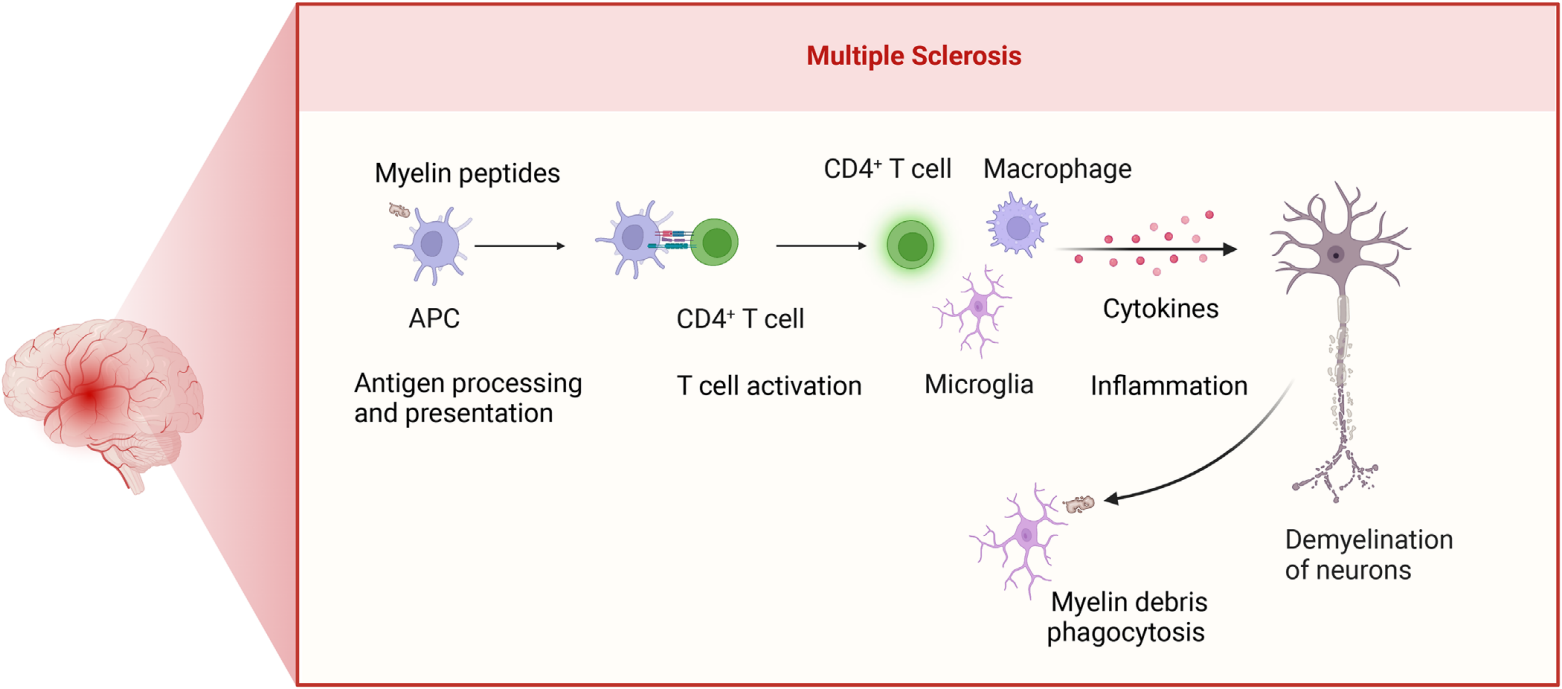
The roles of autophagy dysregulation in the pathogenesis of multiple sclerosis (MS). †Autophagy can act as a two‐edged sword in MS. While inhibiting autophagy may alleviate the inflammatory immune response by interfering with antigen presentation and specific T cell activation, it can also accelerate the accumulation of undesired substrates, such as apoptotic cells, myelin and axonal fragments, and protein aggregates.

MS is a disorder that impacts the central nervous system (CNS) and is distinguished by inflammation and demyelination.^133^ Recent genetic studies have discovered a variant of the autophagy receptor *CALCOCO2/NDP52*, which seems to be linked to autophagy and MS.^134^ In addition, there is abnormal expression of *ATG5* in both the blood and brain tissue of MS patients.^135^, ^136^, ^137^, ^138^ Researchers have utilized mice with autophagy gene knockouts to investigate the role of autophagy in the progression of MS (refer to Table 2). Current existing evidence suggests that the impact of autophagy on MS is multifaceted, with its effects being both advantageous and deleterious, contingent upon the specific cellular context (refer to Figure 4).

**TABLE 2:** The phenotypes of mice with genetically modified autophagy in several autoimmune and inflammatory diseases models.

Disease model	Gene	Strategy of gene editing	Phenotype	References
IBD	*Atg16l1*	Conditional deletion of *Atg16l1* in the IECs	Paneth cell abnormalities; a significant surge in inflammation accompanied by the dissemination of bacteria after infection with *S typhimurium*	^4^
		Knock‐in T300A variant	Goblet cells exhibited impaired granule exocytosis; an insufficient and disrupted mucus layer; diminished mucin secretion; aggravated inflammatory response in DSS‐induced colitis; microbiota dysbiosis	^5^
		Conditional deletion of *Atg16l1* in T cells	Spontaneous intestinal inflammation	^6^
		Knockout *Atg16l1*	Heightened IL‐1β production in response to LPS or endotoxin stimulation; highly susceptible to dextran sulphate sodium‐induced acute colitis	^7^
		Knock‐in T300A variant	Impaired elimination of pathogens; heightened inflammatory cytokine response	^8^
		Knock‐in T300A variant	Abnormal morphology of Paneth cells and goblet cells; increased IL‐1β production	^9^
		Conditional deletion of *Atg16l1* in the IECs	Increased release of proinflammatory cytokines; enhanced apoptosis of intestinal epithelial cells	^10^
		Knock‐in hypomorphic *Atg16l1*	Extraordinary abnormalities in Paneth cells	^93^
	*Atg5*	Conditional deletion of *Atg5* in the IECs	Paneth cell and granule abnormalities	^93^
	*Irgm1*	Knockout *Irgm1*	Significant changes in the positioning and morphology of Paneth cells; heightened acute inflammation after dextran sodium sulfate exposure	^95^
	*Atg4b*	Knockout *Atg4b*	Malfunctions in Paneth cells; heightened vulnerability to colitis induced by DSS	^96^
	*Atg5*	Conditional deletion of *Atg5* in the IECs	Paneth cell morphological abnormalities; significant disruption in the gut microbiota composition accompanied by a decrease in alpha diversity	^100^
	*Atg7*	Conditional deletion of *Atg7* in the colonic epithelial cell	Aggravation of experimental colitis; diminished secretion of colonic mucins; abnormal microflora	^102^
SLE	*Atg5*	Conditional deletion of *Atg5* in B cell	Diminished antibody responses in the context of targeted immunization, parasitic infestation, and inflammation of mucosal tissues; plasma cell differentiation is impaired in B cells lacking *Atg5*	^49^
	*Cybb*	Cross an X‐linked *Cybb* null allele onto MRL.Fas^lpr^ mice	Exacerbated lupus presentation, encompassing heightened splenomegaly, increased renal pathology, and altered profiles of elevated autoantibodies	^110^
	*Ncf1*	Mice with *Ncf1* ^m1j/m1j^ variant on a BALB/c.Q background	Impaired NOX2 complex function; accumulation of secondary necrotic cells in phagocytes of mice, resulting in escalated production of inflammatory mediators	^111^
	*Atg7*	Conditional deletion of *Atg7* in hematopoietic cells (and their progenitors)	Failure of differentiation into plasma cells and moderately decreased viability in *Atg7*‐deficient B cells	^113^
	*Rubcn*	*Rubcn* knockout in MRL.Fas^lpr^ lupus mice	Heightened survival rates, attenuated nephritis, and lowered production of autoantibodies	^115^
		*Rubcn* knockout in B6.Sle1.Yaa lupus mice	Heightened survival rates, reduced nephritis, and decreased autoantibody production	^115^
	*Atg5*	Conditional deletion of *Atg5* in B cell	Impaired antibody response; reduced numbers of antigen‐specific long‐lived plasma cells in the bone marrow; expanded endoplasmic reticulum (ER) size, intensified ER stress signaling and increased death in plasma cells	^116^
		Conditional deletion of *Atg5* in pro‐B cell	Decreased spleen B cells, peritoneal B‐1a and B‐2 B‐cell proportions	^117^
		Conditional deletion of *Atg5* in mature B cell	Decreased spleen B cells and peritoneal B‐2 B cells	^117^
		Conditional deletion of *Atg5* in mature B cell in C57BL/6^lpr/lpr^ autoimmune‐prone mice	Reduced production of antinuclear antibodies, decreased population of long‐lived plasma cells, and diminished accumulation of IgG in renal tissues	^117^
	*Atg7*	Conditional deletion of *Atg7* in B cell	Impaired maintenance of memory B cells after immunization	^118^
	*Atg5*	Conditional deletion of *Atg5* in B cell in *Tlr7.1* transgenic mice	ANA and chronic inflammation did not develop; ameliorated glomerulonephritis; improved overall survival	^125^
		Conditional deletion of *Atg5* in dendritic cells in *Tlr7.1* transgenic mice	Increased lifespan and reduced IFNα level	^126^
		Combined DC and B cell conditional deletion of *Atg5* in *Tlr7.1* transgenic mice	Inflammasome activation and organ damage; splenomegaly and lymphadenopathy; severe lethality	^126^
MS	*Cybb*	Conditional deletion of *Cybb* in conventional dendritic cells	Reduced the severity of EAE	^28^
	*Atg7*	Conditional deletion of *Atg7* in microglia	Progressive MS‐like disease	^141^
		Myeloid‐specific deletion of *Atg7*	Reduce severity of EAE	^144^
	*Pik3c3*	Myeloid‐specific deletion of *Pik3c3*	Reduced severity of EAE	^145^
	*Atg5*	Conditional deletion of *Atg5* in CD11c dendritic cell	Resistance of EAE induction	^146^
	*Atg7*	Conditional deletion of *Atg7* in dendritic cell	Reduce the onset and severity of EAE	^147^
	*Pik3c3*	Conditional deletion of *Pik3c3* in dendritic cell	Decreased occurrence and intensity of EAE	^148^
	*Rb1cc1*	Conditional deletion of *Rb1cc1* in dendritic cell	Resistant to EAE induction	^148^
	*Rubcn*	Conditional deletion of *Rubcn* in dendritic cell	Exhibit symptoms of EAE comparable to wild‐type control mice	^148^
		Knockout *Rubcn*	Exhibit symptoms of EAE comparable to wild‐type control mice	^150^
	*Pik3c3*	Conditional deletion of *Pik3c3* in CD4 cell	Resistance of EAE induction	^150^
	*Becn1*	Conditional deletion of *Becn1* in CD4 cell	Resistance of EAE induction	^151^
	*Atg5*	Conditional deletion of *Atg5* in microglia	Exert no influence on the progression of EAE	^152^

IBD, inflammatory bowel diseases; SLE, systemic lupus erythematosus; MS, multiple sclerosis; IECs, intestinal epithelial cells; DSS, dextran sodium sulfate; LPS, lipopolysaccharide; ANA, antinuclear antibodies; ER, endoplasmic reticulum; NOX2 complex, NADPH oxidase 2 complex; EAE, experimental autoimmune encephalomyelitis; WT, wild type.

#### 4.3.1 Intracellular degradative pathway

In microglia, the primary CNS‐resident macrophages, the machinery of autophagy performs an essential function in regulating phagocytosis, the process of engulfing and digesting unwanted substances, including cellular debris and protein aggregates, to promote tissue repair and homeostasis.^139^, ^140^ Autophagy receptors have been shown to recognize cargos and facilitate subsequent autophagic degradation. LAP, which is the functional convergence of autophagy and phagocytosis, is essential for clearing myelin debris. The gene *Atg7*, which is necessary for both canonical autophagy and LAP, is specifically deleted in the microglia of mice. This deletion leads to the development of a progressive MS‐like disease characterized by impaired autophagy‐associated phagocytosis.^141^ Furthermore, studies conducted on mice with targeted deletion of *Atg5* in microglia have demonstrated that the absence of *Atg5* in these cells results in increased intracellular accumulation of myelin and inflammatory debris. This accumulation subsequently contributes to persistent neuroinflammation and neurotoxicity.^142^, ^143^ However, mice with microglial *Ulk1* specifically deleted, a gene required only for canonical autophagy, did not exhibit a similar disease phenotype, suggesting that the disease phenotype was mainly dependent on noncanonical autophagy rather than canonical autophagy.

#### 4.3.2 Antigen processing and presentation

Experimental autoimmune encephalomyelitis (EAE) is an extensively used animal model to investigate MS, and many investigations have shown that modulation of autophagy leads to a significant improvement in the severity of EAE. Autophagy participates in the regulation of antigen loading onto MHC molecules and the subsequent antigen presentation by APCs, including dendritic cells and macrophages. Selective deletion of *Atg7* or *Pik3c3* specifically in myeloid cells of mice leads to a decrease in EAE severity,^144^, ^145^ indicating the therapeutic prospects of autophagy for EAE treatment. Additionally, selective deficiency of *Pik3c3*, *Rb1cc1/Fip200*, *Atg5*, or *Atg7* in dendritic cells leads to a reduced occurrence and gravity of EAE,^146^, ^147^, ^148^, ^149^ primarily attributed to diminished handling and display of self‐antigens by APCs to CD4 T lymphocytes. Of note, mice lacking *Rubcn*, a gene essential for LAP but not canonical autophagy, in dendritic cells, displayed wild‐type levels of EAE susceptibility, which differs significantly from DC‐specific *Pik3c3*‐deficient or *Rb1cc1*‐deficient mice.^148^ These findings suggest that the autophagy defect of these cells, rather than LAP, is responsible for EAE resistance, establishing a connection between autophagy and the processing of antigens as well as the pathogenicity of autoimmune T cells.

#### 4.3.3 Activation and survival of T cells

The hallmark of MS lies in the activation and proliferation of autoreactive CD4 T cells.^138^ Research findings suggest that inhibiting autophagy can reduce the severity of EAE in animal models by regulating the activation and survival of autoreactive T cells. Specifically, mice with a T cell‐specific deletion of *Pik3c3* or *Becn1* displayed resistance to EAE induction and exhibited impaired ability to induce autoreactive T cell responses.^150^, ^151^ One underlying cause for this observation is the disrupted energy metabolism and functional dynamics of T cells, wherein autophagy is indispensable for supporting ATP production during the activation of T cells. As an exemplification, T cells lacking *Pik3c3* exhibited suppressed oxidative respiration and diminished glycolysis,^150^ leading to a failure in T helper 1 cell differentiation. Additionally, the maintenance of cell viability in activated T cells relies on autophagy, which facilitates the degradation of apoptotic proteins like Bim and Bcl‐2, effectively suppressing cellular apoptosis. Indeed, *Becn1*‐deficient T cells experience a significant increase in cell death upon stimulation.^151^ Therefore, the modulation of autophagy in T cells presents a promising therapeutic strategy for MS.

## 5 THE THERAPEUTIC POTENTIAL OF TARGETING AUTOPHAGY

Autophagy‐targeted drugs and biological agents have shown great potential as effective therapeutic options in autoimmune and inflammatory diseases. Several drugs have been used in clinical treatment, including rapamycin and hydroxychloroquine (HCQ). Clinical trials investigating various autophagy modulators are summarized in Table 3. Rapamycin and its analog, which are mTORC1 pathway inhibitors, can induce autophagy and have shown potential clinical efficacy in SLE based on several clinical trials.^153^, ^154^, ^155^, ^156^ A phase 1/2 open‐label prospective clinical trial provided compelling evidence that rapamycin exhibits safety, excellent tolerability, and significant clinical effectiveness in patients with SLE.^153^ After 12 months of sirolimus therapy, a steady amelioration in disease activity manifested among patients diagnosed with active SLE. Moreover, there is supportive data for the efficacy of using rituximab to improve renal outcomes in patients with LN.^155^, ^156^ This includes notable reductions in proteinuria levels. Vitamin D, another autophagy inducer, have also been associated with the regulation of autoimmune diseases.^157^, ^158^, ^159^, ^160^ Autophagy inhibitors like chloroquine/HCQ, which raise the pH level within lysosomes and hinder the fusion between autophagosomes and lysosomes,^161^ have been proposed as promising therapeutic choices with immunomodulatory effects for rheumatoid arthritis.^162^ In addition to pharmacological modulators, peptide‐based agents are a novel therapeutic option. The evaluation of a Phase III trial is underway for a novel phosphopeptide, known as Lupuzor (P140), which exhibits autophagy‐inhibiting properties. A previous phase IIb clinical trial confirmed its safety and tolerability,^163^ and Lupuzor demonstrated remarkable efficacy in reducing disease activity among individuals with a SLEDAI score of 6 or higher, presenting encouraging outcomes in advancing the treatment of lupus.

**TABLE 3:** Several autophagy‐targeted drugs and biological agents and relevant clinical trials in inflammatory bowel diseases (IBD), systemic lupus erythematosus (SLE), and multiple sclerosis (MS).

Agent	Disease	Trial ID	Status	Study title	Intervention/treatment	References
Sirolimus (or rapamycin)—autophagy inducer	IBD	NCT00392951	Completed (Has Results)	Sirolimus for Autoimmune Disease of Blood Cells	Drug: sirolimus	NA
		NCT02675153	Recruiting	To Evaluate the Efficacy and Safety of Rapamycin for Crohn's Disease‐related Stricture	Drug: rapamycin	NA
		NCT04691232	Recruiting	Autologous Ex Vivo Expanded Regulatory T Cells in Ulcerative Colitis	Biological: autologous, ex vivo expanded, regulatory T cells	NA
	SLE	NCT00779194	Completed	Prospective Study of Rapamycin for the Treatment of SLE	Drug: rapamycin	^153^
		NCT04582136	Not yet recruiting	Efficacy and Safety of Sirolimus in Active Systemic Lupus Erythematosus	Drug: sirolimus Drug: placebo	NA
		NCT04736953	Not yet recruiting	Sirolimus Treatment Of Patients With SLE	Drug: sirolimus Other: placebo	NA
	MS	NCT00095329	Terminated	Treating Multiple Sclerosis With Sirolimus, an Immune System Suppressor	Biological: sirolimus	NA
Metformin—autophagy inducer	IBD	NCT05553704	Not yet recruiting	Metformin in Patients With Ulcerative Colitis Treated With Mesalamine	Drug: metformin	NA
		NCT04750135	Not yet recruiting	Assessment of Metformin as Adjuvant Therapy in Patients With Ulcerative Colitis	Drug: metformin 500 mg TID oral tablet Drug: placebo	NA
	SLE	NCT02741960	Completed	The Effect of Metformin on Reducing Lupus Flares	Drug: metformin Drug: placebo	^168^
	MS	NCT05349474	Recruiting	Metformin Treatment in Progressive Multiple Sclerosis	Drug: metformin 500 mg oral tablet, up to four tablets a day Drug: placebo oral tablet identical to metformin, up to four tablets a day	NA
		NCT04121468	Recruiting	A Phase I Double Blind Study of Metformin Acting on Endogenous Neural Progenitor Cells in Children With Multiple Sclerosis	Drug: metformin Other: placebo	NA
		NCT05131828	Recruiting	CCMR Two: A Phase IIa, Randomised, Double‐blind, Placebo‐controlled Trial of the Ability of the Combination of Metformin and Clemastine to Promote Remyelination in People With Relapsing‐remitting Multiple Sclerosis Already on Disease‐modifying Therapy	Drug: metformin and clemastine in combination Drug: placebo	NA
		NCT05298670	Recruiting	Drug Repurposing Using Metformin for Improving the Therapeutic Outcome in Multiple Sclerosis Patients	Drug: metformin 1000 mg oral tablet Drug: interferon beta‐1a	NA
Vitamin D—autophagy inducer	IBD	NCT00742781	Completed (Has Results)	Vitamin D Supplementation in Crohn's Patients	Dietary supplement: vitamin D	NA
		NCT00621257	Terminated (Has Results)	Vitamin D Levels in Children With IBD	Dietary supplement: ergocalciferol Dietary supplement: cholecalciferol	^158^, ^169^
		NCT02208310	Terminated (Has Results)	Trial of High Dose Vitamin D in Patient's With Crohn's Disease	Drug: cholecalciferol 10,000 IU Drug: cholecalciferol 400 IU	^170^
		NCT02076750	Completed	Weekly Vitamin D in Pediatric IBD	Dietary supplement: vitamin D3 (cholecalciferol)	NA
		NCT01877577	Completed	Supplementation of Vitamin D3 in Patients With Inflammatory Bowel Diseases and Hypovitaminosis D	Dietary supplement: vitamin D3	NA
		NCT02615288	Completed	High Dose Vitamin D3 in Crohn's Disease	Dietary supplement: vitamin D3	^171^
		NCT01692808	Completed	Bioavailability of Vitamin D in Children and Adolescents With Crohn's Disease	Drug: vitamin D3 3000 UI daily Drug: vitamin D3 4000 UI daily	^172^
		NCT01369667	Completed	Vitamin D Supplementation in Adult Crohn's Disease	Dietary supplement: vitamin D3 Other: placebo	^173^
		NCT02186275	Completed	The Vitamin D in Pediatric Crohn's Disease	Drug: vitamin D3: 3000 or 4000 UI/day then 2000 UI/day Drug: vitamin D3 800 UI/day then 800 UI/day	NA
		NCT01187459	Completed	Vitamin D in Pediatric Crohn's Disease	Dietary supplement: vitamin D	^174^
		NCT01864616	Terminated	The Impact of Vitamin D on Disease Activity in Crohn's Disease	Dietary supplement: vitamin D3	NA
		NCT03615378	Terminated	Maintenance Dosing of Vitamin D in Crohn's Disease	Dietary supplement: 5000 IU D3 Dietary supplement: 1000 IU D3 Dietary supplement: placebo	NA
		NCT01046773	Terminated	Vitamin D Supplementation as Nontoxic Immunomodulation in Children With Crohn's Disease	Drug: cholecalciferol	NA
		NCT04331639	Recruiting	High Dose Interval Vitamin D Supplementation in Patients With Inflammatory Bowel Disease Receiving Biologic Therapy	Dietary supplement: vitamin D3	NA
		NCT04828031	Recruiting	Vitamin D Regulation of Gut Specific B Cells and Antibodies Targeting Gut Bacteria in Inflammatory Bowel Disease	Drug: vitamin D	NA
		NCT03999580	Recruiting	The Vitamin D in Pediatric Crohn's Disease (ViDiPeC‐2)	Drug: vitamin D3	NA
		NCT04225819	Recruiting	Adjunctive Treatment With Vitamin D3 in Patients With Active IBD	Dietary supplement: vitamin D3 Other: placebo	NA
		NCT04991324	Not yet recruiting	Cholecalciferol Comedication in IBD—The 5C‐study	Drug: vitamin D3	NA
		NCT04308850	Not yet recruiting	Exploring the Effects of Vitamin D Supplementation on the Chronic Course of Patients With Crohn's Disease With Vitamin D Deficiency	Drug: vitamin D drops	NA
	SLE	NCT00710021	Completed (Has Results)	Vitamin D3 in Systemic Lupus Erythematosus	Drug: vitamin D3 Drug: vitamin D3 placebo	^175^
		NCT01911169	Completed (Has Results)	Vitamin D to Improve Endothelial Function in SLE	Drug: cholecalciferol	NA
		NCT01709474	Terminated (Has Results)	Vitamin D3 Treatment in Pediatric Systemic Lupus Erythematosus	Drug: vitamin D3 6000 IU Drug: vitamin D3 400 IU	NA
		NCT05430087	Completed	Vitamin D and Curcumin Piperine Attenuates Disease Activity and Cytokine Levels in Systemic Lupus Erythematosus Patients	Drug: cholecalciferol (vitamin d3) and curcumin‐piperine.	NA
		NCT01892748	Completed	Cholecalciferol Supplementation on Disease Activity, Fatigue and Bone Mass on Juvenile Systemic Lupus Erythematosus.	Drug: cholecalciferol Drug: placebo	^160^, ^176^
		NCT00418587	Completed	Vitamin D Therapy in Patients With Systemic Lupus Erythematosus (SLE)	Drug: cholecalciferol	NA
		NCT01425775	Completed	The Effect of Vitamin D Supplementation on Disease Activity Markers in Systemic Lupus Erythematosus (SLE)	Drug: vitamin D 25(OH)D Other: placebo	NA
		NCT05326841	Completed	Effect of Cholecalciferol Supplementation on Disease Activity and Quality of Life of Systemic Lupus Erythematosus Patients	Drug: vitamin D3	NA
		NCT03155477	Completed	Effect Of Curcuma Xanthorrhiza and Vitamin D3 Supplementation in SLE Patients With Hypovitamin D	Dietary supplement: “cholecalciferol” and “C. Xanthorrhiza” Dietary supplement: “cholecalciferol” and “placebo”	^177^
		NCT05260255	Recruiting	Effect of Vitamin D Supplement on Disease Activity in SLE	Drug: vitamin D2 (calciferol) Drug: placebo	NA
		NCT05392790	Recruiting	Progressive Resisted Exercise Plus Aerobic Exercise on Osteoporotic Systemic Lupus Erythmatosus	Other: progressive resisted exercise training Drug: calcium and Vit D Other: aerobic exercises	NA
	MS	NCT00940719	Completed	Vitamin D3 Supplementation and the T Cell Compartment in Multiple Sclerosis (MS)	Dietary supplement: vitamin D3	^159^
		NCT01667796	Completed (Has Results)	Pharmacokinetics of Vitamin D in Multiple Sclerosis and in Health	Dietary supplement: vitamin D3	^178^
		NCT01490502	Completed (Has Results)	Vitamin D Supplementation in Multiple Sclerosis	Drug: vitamin D3	^179^
		NCT00644904	Completed	Safety Trial of High Dose Oral Vitamin D3 With Calcium in Multiple Sclerosis	Dietary supplement: vitamin D3	^180^
		NCT01257958	Completed	Vitamin D Pilot Study in Patients With Multiple Sclerosis	Drug: vitamin D	NA
		NCT01024777	Completed	Safety and Immunologic Effect of Low Dose Versus High Dose Vitamin D3 in Multiple Sclerosis	Drug: cholecalciferol	NA
		NCT03385356	Completed	Impact of Vitamin D Supplementation in Patients With Multiple Sclerosis	Drug: vitamin D	NA
		NCT02696590	Completed	High Dose Oral Versus Intramuscular Vitamin D3 Supplementation In Multiple Sclerosis Patients	Dietary supplement: vitamin D3	NA
		NCT00785473	Completed	Can Vitamin D Supplementation Prevent Bone Loss in Persons With Multiple Sclerosis	Dietary supplement: cholecalciferol Dietary supplement: calcium carbonate	^181^
		NCT01440062	Terminated	Efficacy of Vitamin D Supplementation in Multiple Sclerosis (EVIDIMS)	Drug: verum arm receiving vitamin D oil Drug: low dose arm receiving neutral oil and 400 IU/g of vitamin D every second day	^182^
		NCT03610139	Recruiting	Longitudinal Effect of Vitamin D3 Replacement on Cognitive Performance and MRI Markers in Multiple Sclerosis Patients	Dietary supplement: vitamin D3	NA
		NCT05340985	Not yet recruiting	Investigating the Effects of Hydroxyvitamin D3 on Multiple Sclerosis	Dietary supplement: 25(OH)D3 Dietary supplement: vitamin D3	NA
Lupuzor/P140 peptide—autophagy inhibitor	SLE	NCT02504645	Completed (Has Results)	A 52‐Week, Randomized, Double‐Blind, Parallel‐Group, Placebo‐Controlled Study to Evaluate the Efficacy and Safety of a 200‐mcg Dose of IPP‐201101 Plus Standard of Care in Patients With Systemic Lupus Erythematosus	Drug: lupuzor Drug: placebo Other: standard of care	NA
		EudraCT number:2007‐004892‐21	Completed	Lupuzor/P140 Peptide in Patients with Systemic Lupus Erythematosus: A Randomised, Double‐Blind, Placebo‐Controlled Phase IIb Clinical Trial	Drug: lupuzor Drug: placebo	^163^
		No. 143/14.06.2006 (Bulgaria)	Completed	Spliceosomal Peptide P140 for Immunotherapy of Systemic Lupus Erythematosus	Drug: lupuzor Other: standard of care	^183^
		NCT01135459	Completed	A Study to Evaluate the Efficacy and Safety of CEP‐33457 in Patients With Systemic Lupus Erythematosus	Drug: lupuzor Drug: placebo	NA
		NCT01240694	Terminated	A Long‐Term Study of the Safety and Tolerability of Repeated Administration of CEP‐33457 in Patients With Systemic Lupus Erythematosus	Drug: lupuzor	NA
Chloroquine/hydroxychloroquine (HCQ)—autophagy inhibitor	IBD	NCT01783106	Completed	Antibiotics and Hydroxychloroquine in Crohn's	Drug: ciprofloxacin Drug: doxycycline Drug: hydroxychloroquine Drug: budesonide	^184^
		NCT05119140	Recruiting	HCQ for Non‐Europeans With Mild to Severe UC	Drug: hydroxychloroquine Drug: mesalamine	NA
	SLE	NA	Completed	Hydroxychloroquine in Lupus Pregnancy	No HCQ exposure during pregnancy; continuous use of HCQ during pregnancy; cessation of HCQ treatment either in the 3 months prior to or during the first trimester of pregnancy	^185^
		NCT00413361	Completed	The Reduction of Systemic Lupus Erythematosus Flares: Study PLUS	Drug: versus hydroxychloroquine	^186^
		NCT01551069	Completed	Multicenter Study Assessing the Efficacy & Safety of Hydroxychloroquine Sulfate in Patients With Systemic Lupus Erythematosus or Cutaneous Lupus Erythematosus With Active Lupus Erythematosus Specific Skin Lesion	Drug: hydroxychloroquine (Z0188) Drug: placebo	^187^
		NCT03019926	Completed	Lupus and Observance	Biological: hydroxychloroquine dosage	NA
		NCT02842814	Completed	Prediction of Relapse Risk in Stable Systemic Lupus Erythematosus	Other: drug free Drug: HCQ Drug: GC+HCQ	NA
		NCT01946880	Terminated (Has Results)	Randomized MMF Withdrawal in Systemic Lupus Erythematosus (SLE)	Drug: mycophenolate mofetil Drug: hydroxychloroquine or chloroquine Drug: prednisone	NA
		NCT03802188	Recruiting	Hydroxychloroquine in Systemic Lupus Erythematosus	Observational study	NA
		NCT03030118	Active, not recruiting	Study of Anti‐Malarials in Incomplete Lupus Erythematosus	Drug: hydroxychloroquine Drug: placebo oral capsule	
		NCT03802188	Recruiting	Hydroxychloroquine in Systemic Lupus Erythematosus	Observational study	NA
	MS	NCT02913157	Completed	Hydroxychloroquine in Primary Progressive Multiple Sclerosis	Drug: hydroxychloroquine	^188^
		NCT05013463	Recruiting	Hydroxychloroquine and Indapamide in SPMS	Drug: hydroxychloroquine pill Drug: indapamide pill	NA

Data were collected from the clinical registration website (http://clinicaltrials.gov/); IBD, inflammatory bowel diseases; SLE, systemic lupus erythematosus; MS, multiple sclerosis; NA, not available.

Moreover, recent investigations have focused on the modulation of autophagy to enhance immune responses and facilitate antitumor effects. Clinical trials targeting autophagy, either alone or in combination, in cancer treatment have mostly relied on HCQ, and the outcomes of these trials have been summarized in some recent reviews.^51^, ^54^, ^164^, ^165^ Despite some patients showing partial response or prolonged stable disease, overall response and survival rates have not been improved, leading to the exploration of more specific and targeted therapies. Due to its capacity to regulate the immune response against tumors, targeting autophagy has emerged as a promising strategy in the realm of tumor immunotherapy. For example, in a pancreatic cancer model, the combination of chloroquine‐mediated autophagy inhibition and the administration of immune checkpoint blockade (ICB) therapy targeting PD‐1 and CTLA‐4 has synergistically boosted the antitumor immune response.^56^ By efficiently inhibiting tumor‐specific autophagy, there is a notable increase in antigen presentation, resulting in heightened responsiveness of PDAC to dual ICB. Furthermore, the implementation of this strategy enhances the infiltration of CD8+ T cells and facilitates the eradication of tumor cells. In addition to autophagy inhibitors, the induction of autophagy also holds promise for enhancing the efficacy of tumor immunotherapy. Studies have revealed the remarkable potential of a pH‐sensitive nanocarrier, which effectively regulates autophagy, in significantly augmenting the immunotherapeutic response during the treatment of osteosarcoma. This nanocarrier, when combined with PD‐1/PD‐L1 blockade, exhibits promising outcomes in enhancing the efficacy of immunotherapy.^166^ Employing nanoparticles as a strategy to manipulate autophagy represents a promising approach for cancer treatment. These nanoparticles, serving as effective delivery systems, offer potential in leveraging autophagy for therapeutic applications.^167^

While certain drugs mentioned above, including rapamycin and HCQ, have been approved for human use, their clinical application was not specifically targeted toward autophagy regulation.^24^ Additionally, the extent to which the pharmacological effects rely on autophagy remains uncertain. Therefore, significant efforts are still required to advance the development of clinically viable interventions targeting autophagy. It is imperative to conduct in‐depth mechanistic investigations to elucidate the precise role of autophagy. Moreover, the development of autophagy modulators with specificity and the precise regulation of autophagic flux are crucial in order to maintain a delicate balance within autophagy processes, as excessive autophagy activity can also be harmful to cells.

## 6 CONCLUSIONS AND FUTURE DIRECTIONS

This review emphasizes the essential role of autophagy in both innate and adaptive immune responses, encompassing crucial functions such as clearance of pathogens, processing and presentation of antigens, production of cytokines, and the survival and differentiation of lymphocytes. Malfunctioning of autophagy in humans can lead to various immune disorders, such as inflammatory, autoimmune or general immunity disorders. Inhibiting autophagy can alleviate diseases like SLE and MS by interfering with antigen presentation and lymphocyte survival, but it may also exacerbate diseases such as IBD. Dysfunctional autophagy can disrupt intestinal epithelial homeostasis, change gut microbiota composition, and amplify intestinal inflammation in IBD. These findings demonstrate that autophagy regulation varies across different diseases, tissues, and cells. Therefore, Therefore, future investigations should focus on elucidating the distinct roles of autophagy in different tissues and cell types, aiming to gain a comprehensive understanding of disease‐specific mechanisms.

While many autophagy modulators are currently undergoing clinical trials, there are still several unresolved clinical questions. First, current pharmacological agents like rapamycin and HCQ indirectly modulate autophagy, and there is a lack of approved pharmaceuticals that specifically address autophagy as a therapeutic target. Second, the development of targeted therapies necessitates not only a drug with exceptional specificity but also a comprehensive understanding of the optimal timing and administration protocols. Methods for accurately quantifying the activity of the autophagy pathway in vivo are currently inadequate. Consequently, assessing the extent of autophagy modulation in patients undergoing therapy with autophagy modulators presents a significant challenge. Last, despite the promising therapeutic opportunities that autophagy has presented for effective cancer treatment, significant challenges and obstacles exist. Autophagy in tumor development exhibits a complex and multifaceted nature, encompassing both advantageous and detrimental contributions. Although the suppression of autophagy in certain scenarios can bolster the immune response and bolster the antitumor effects of immunotherapy, it can also contribute to immune evasion by tumor cells and foster resistance to treatment. Therefore, identifying the dominant role of autophagy (antitumorigenic or protumorigenic) in particular cancers and deciding when to target autophagy for treatment during tumor development are crucial questions.

In conclusion, autophagy is integral to the maintenance of immune homeostasis and serves as a protective mechanism against inflammatory and autoimmune diseases. The suppression or enhancement of autophagy has been shown to have significant effects in various cell culture and animal models, impacting self‐immune responses, inflammatory conditions, and a variety of tumors. These discoveries have provided invaluable knowledge regarding the underlying mechanisms of these diseases and have paved the way for the emergence of groundbreaking therapeutic approaches. For instance, research has shown that effectively inhibiting autophagy effectively reduces the onset and severity of actively induced EAE, providing a compelling intervention strategy for autoimmune diseases informed by an animal model.^148^ Furthermore, it may be crucial to identify suitable patients for personalized therapy by considering both disease pathology and genotype, such as individuals with IBD who possess the *ATG16L1* ^T300A/T300A^ genotype. In addition to conventional treatment options, gene therapy emerges as a potential avenue for tackling IBD. Excitingly, several miRNAs have been discovered to effectively suppress autophagy.^189^ Therefore, it is essential to develop more selective autophagy modulators, more reliable methods of quantifying autophagic activity in vivo, and more personalized therapeutic strategies.

## AUTHOR CONTRIBUTION

Ting Gan conceptualized this review and drafted the manuscript. Shu Qu drew the figures and edited the language. Xu‐jie Zhou and Hong Zhang provided helpful suggestions on the structure and content of this review. All authors revised the manuscript. All authors have read and approved the article.

## CONFLICT OF INTEREST STATEMENT

All authors have approved of the manuscript and declare no potential conflict of interest.

## ETHICS STATEMENT

Not applicable.

## Data Availability

All the presented information in this article is accessible by contacting the corresponding author.
